# Outcomes of patients with spinal metastases from renal cell carcinoma treated with conventionally-fractionated external beam radiation therapy

**DOI:** 10.1097/MD.0000000000019838

**Published:** 2020-04-17

**Authors:** Chia Ching Lee, Jeremy Chee Seong Tey, Timothy Cheo, Chau Hung Lee, Alvin Wong, Naresh Kumar, Balamurugan Vellayappan

**Affiliations:** a Department of Radiation Oncology, National University Cancer Institute, Singapore; National University Hospital, Singapore; National University Health System, Singapore; National University of Singapore; b Department of Diagnostic Radiology, Tan Tock Seng Hospital; c Department of Medical Oncology, National University Cancer Institute; d Department of Orthopaedic Surgery, National University Hospital, Singapore; National University Health System, Singapore; National University of Singapore, Singapore.

**Keywords:** conventionally-fractionated external beam radiotherapy, metastases, renal cell carcinoma, spine

## Abstract

Renal cell carcinoma (RCC) has been traditionally thought to be radioresistant. This retrospective cohort study aims to assess the outcomes of patients with spinal metastases from RCC treated with conventionally-fractionated external beam radiation therapy (cEBRT) in our institution.

Patients diagnosed with histologically or radiologically-proven RCC who received palliative cEBRT to spinal metastases, using 3-dimensional conformal technique between 2009 and 2018 were reviewed. Local progression-free survival (PFS), overall survival (OS) and common terminology criteria for adverse events version 4.0-graded toxicity were assessed. Univariable and multivariable Cox proportional hazards regression analyses were performed to evaluate for predictors associated with survivals.

Thirty-five eligible patients with forty spinal segments were identified, with a median follow-up of 7 months (range, 0–47). The median equivalent dose in 2 Gy fractions (EQD_2_) was 32.5 Gy _10_ (range, 12–39). Thirty-seven percent of patients underwent surgical intervention. At the time of last follow-up, all but 1 patient had died. Seven patients developed local progression, with the median time to local progression of 10.2 months. The median local PFS and OS were 3.3 and 4.8 months. There was no grade 3 or higher toxicity. A higher radiation dose (equivalent dose to 2 Gy fraction <32.5 Gy _10_ vs ≥32.5Gy _10_) (hazard ratio [HR], 0.47; 95% confidence interval [CI], 0.17–3.18; *P*-value (*P*) = .68) and spinal surgery (HR, 2.35; 95% CI, 0.53–10.29; *P* = .26) were not significantly associated with local PFS on univariable analysis. Multivariable analysis showed that higher Tokuhashi score (HR, 0.41; 95% CI, 0.19–0.88; *P* = .02), lower number of spinal segments irradiated (HR, 1.18; 95% CI, 1.01–1.37; *P* = .04) and use of targeted therapy (HR, 0.41; 95% CI, 0.18–0.96; *P* = .04) were independent predictors for improved OS.

For an unselected group of patients with RCC, there is no significant association between higher radiation dose and improved local control following cEBRT. This may be due to their short survivals. With the use of more effective systemic therapy, including targeted therapy and immunotherapy, survival will likely be prolonged. A tailored-approach is needed to identify patients with good prognosis who may still benefit from aggressive local treatments.

## 1 Introduction

Renal cell carcinoma (RCC) constitutes approximately 85% of malignant neoplasms of the kidney and 3% of all new cancer cases.^[1,2]^ Osseous metastases occur in 30% of patients with RCC, with the vertebra being the most common site.^[3]^ Spinal metastases can impact the quality of life when they are complicated by intractable pain, spinal cord compression with resultant neurological deficits and pathological fractures. Radiotherapy (RT) is widely used in the palliative treatment of spinal metastases.

RCC poses an interesting challenge in oncologic treatment as it is a highly vascularized neoplasm and historically thought to be resistant to both RT and chemotherapy. It has been shown on both in vitro and clinical studies that RCC is radioresistant to conventionally-fractionated external beam radiation therapy (cEBRT).^[4–6]^ Previous studies done in 1980s described the response rates of cEBRT in metastatic RCC ranging from 30% to 80%,^[7–10]^ but the applicability of these findings in this era of modern RT techniques and novel targeted therapy is unclear. In recent years, stereotactic body radiation therapy (SBRT) is increasingly utilized for the treatment of RCC because it is believed that ultra-high dose per fraction is able to overcome its radioresistant nature and leads to endothelial and microvascular damage in the highly vascularized renal neoplasms.^[11]^ Based on current available literature, SBRT appears to be a promising treatment approach which provides better local control and pain relief although randomized controlled trials comparing SBRT and cEBRT are lacking.^[12–15]^ However, SBRT may not be feasible in certain circumstances, for instance when there is spinal cord compression, diffuse spinal disease and widely metastatic and/ or rapidly progressive disease with limited life expectancy.^[12,16]^

Despite the emerging role of SBRT, cEBRT remains an important treatment option of spinal metastases in RCC. There is a paucity of evidence on the outcomes of contemporary cEBRT in this population.^[11]^ Hence, we performed a retrospective study and aimed to evaluate the outcomes of patients with spinal metastases from RCC treated with cEBRT using our local institutional data.

## 2 Methods

### 2.1 Study design

This is a retrospective cohort study approved by the institutional review board.

### 2.2 Study population

**Figure 1:**
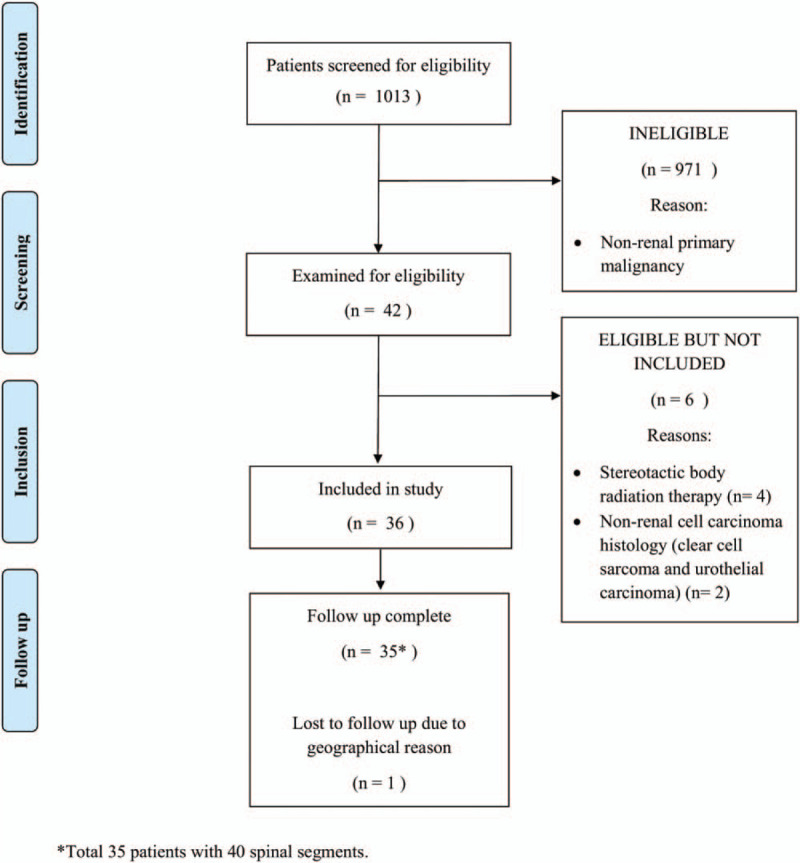
STROBE flow diagram.

We screened the institutional RT database for patients who had received palliative RT for spinal metastases. Patients diagnosed with histologically- or radiological-proven RCC who received palliative cEBRT to spinal metastases using 3-dimensional conformal radiation therapy technique between January 2009 and June 2018 in our institution were included. Those who were treated using SBRT were excluded. The STROBE flow diagram is illustrated in Figure 1.

### 2.3 Radiation therapy

In our institution, RT was offered for the following indications:

(1) palliation of pain,
(2) spinal cord or nerve root compression, and
(3) in the postoperative setting.

RT was planned using computed tomography (CT) simulation. The involved vertebral segments were localized with reference to diagnostic CT and/or magnetic resonance imaging (MRI) imaging. The RT portal included any soft tissue component with an adequate margin, and at least 1 vertebral level above and below the affected segments. Radiation planning was done via XiO planning system, with at least 85% of the prescribed dose covering the entire vertebral body. RT was delivered using a 6 to 10-megavoltage posterior field; an additional lightly-weighted anterior field (eg, anteroposterior-posteroanterior technique) was utilized in patients where the maximum dose exceeded 125% of the prescribed dose. The most common dose fractionation regimens for spinal metastases used in our department were 30 Gy in 10 fractions and 20 Gy in 5 fractions delivered over consecutive weekdays. The dose fractionation regimen was determined upon the discretion of treating radiation oncologists based on the clinical factors, performance status, and life expectancy. Quality assurance of the radiation volumes and plans was performed within the first week of starting RT.

For patients who were on systemic therapy such as targeted therapy, they were instructed to withhold during RT period.

### 2.4 Co-variates

Clinical data were obtained from electronic medical record and RT database. Sociodemographic characteristics were collected: age at diagnosis, gender, ethnicity, Eastern Cooperative Oncology Group (ECOG) performance status (0–4) and Charlson comorbidity index. Tumor characteristics were also gathered: histologic subtypes (clear cell or nonclear cell), Fuhrman grading, International Metastatic RCC Database Consortium (IMDC)/Heng risk model, revised Tokuhashi score, spinal instability neoplastic score (SINS), Bilsky grade, presence of soft tissue mass, visceral metastases and pre-treatment imaging using MRI. In addition, treatment details were also recorded: radiation dose fractionation (the delivered rather than the prescribed dose), equivalent dose to 2 Gy fraction (EQD_2_) assuming an alpha-beta ratio of 10, number of spinal levels irradiated, spinal surgery (decompression in the form of laminectomy and/ or corpectomy, stabilization only or no surgery), nephrectomy, use of tyrosine kinase therapy, anti-vascular endothelial growth factor receptor, immunotherapy, and bone-modifying agents.

Charlson comorbidity index is a validated tool used to predict 10-year survival in patients with multiple comorbidities.^[17,18]^ Fuhrman grading is a commonly-used histologic grading system for survival prognostication.^[19,20]^ IMDC risk model, also known as Heng model, is a reliable tool to guide the selection of systemic treatment including targeted therapy and immunotherapy in the patients for first-line treatment of metastatic RCC.^[21–23]^ IMDC risk model was chosen for our study instead of previously widely-used Memorial Sloan-Kettering Cancer Center (MSKCC)/Motzer score because 2 additional adverse prognostic factors (absolute neutrophil and platelet counts) were added to the 4 factors originally identified by MSKCC (time from diagnosis to systemic therapy, Karnofsky performance status, hemoglobin, corrected calcium) and this was externally validated. IMDC risk model classifies patients into 3 different risk groups (good, intermediate, and poor risk) which have significantly different overall survivals (OSs) and response towards systemic treatment. The revised Tokuhashi scoring system is used as preoperative evaluation which categorizes patients into 3 groups of different scores (0–8, 9–11, and 12–15) with different life expectancy (less than 6 months, 6–12 months and more than 12 months) and guides to propose different treatment options varying from palliative management to decompression procedures with or without fixation, up to radical en bloc excision.^[24]^ The scoring system is based on 6 parameters comprising of Karnofsky performance status, the number of extra-spinal bone metastases, the number of vertebral metastases, the number of metastases to the major internal organs, primary site of cancer, and the presence of palsy.^[24]^ SINS is a tool used to assess spinal stability which is comprised of 5 components, including location, mechanical pain, bone lesion, radiographic spinal alignment, vertebral body collapse, and posterolateral involvement.^[25]^ A score of 0 to 6 is considered as stable; 7 to 12 as potentially unstable; and 13 to 18 as unstable.^[25]^ Bilsky grade is a 6-point, MRI-based grading system used to determine the degree of epidural spinal cord compression.^[26]^ The higher Bilsky grade indicates the more severe spinal cord compression. The presence of soft tissue mass was defined as cortical breach of vertebral body on CT or MRI. The SINS score, Bilsky grade, and presence of soft tissue mass was determined by reviewing radiological reports and imaging and, if clarification was required, in consultation with the reporting radiologist.

EQD_2_ was calculated using the linear quadratic equation: total dose ([dose per fraction + alpha/beta]/[2 Gy + alpha/beta]).

### 2.5 Outcomes

The outcomes of interest in this study were local progression-free survival (PFS), OS, pain response, and toxicity. Local progression was defined as clinical and/or radiological (CT or MRI) progression or events that warranted further local treatment in the form of either re-irradiation or surgery. Radiological progression was assessed using the response evaluation criteria in solid tumors guideline version 1.1.^[27]^ Local PFS was calculated from the start of RT to local progression, death from any cause or last follow-up. OS was calculated from the start of RT to the date of death from any cause or last follow-up. Pain response was defined as complete response, partial response and no response. Toxicity was assessed and graded using common terminology criteria for adverse events version 4.0. Patients were reviewed clinically at 1-month post-RT and 3 to 6 monthly thereafter.

### 2.6 Statistical analysis

The follow-up interval was calculated from start of RT to the date of last censor or death. Frequency with percentage and median with range were used to describe the baseline characteristics of this study cohort. The age at diagnosis, SINS, Bilsky grade and number of spinal levels irradiated were analyzed as continuous variables. ECOG performance status (0–1 vs 2–4), Charlson comorbidity index (<9 vs ≥9), IMDC risk score (intermediate and good vs poor risk), revised Tokuhashi score (<7 vs ≤7), presence of soft tissue mass (yes vs no), presence of visceral metastases (yes vs no), EQD_2_ of ≥32.5 Gy_10_ (vs <32.5 Gy_10_), spinal surgery (yes vs no), nephrectomy (yes vs no), and use of tyrosine kinase inhibitor (yes vs no) were analyzed as dichotomous variables. Survival analyses were plotted on Kaplan–Meier curves. Univariable and multivariable Cox proportional hazards regression were performed to look for the factors that were associated with local PFS and OS. Local PFS was analyzed with death from any cause as the competing event. The variables with *P*-value of less than .10 on univariable analyses were entered into multivariable models to identify predictors for local PFS and OS. *P*-value of less than .05 was considered as statistically significant. The analyses were performed using STATA version 14.

## 3 Results

### 3.1 Baseline characteristics

Baseline characteristics of the study population are summarized in Table 1 . Thirty-five eligible patients with 40 spinal segments were identified. Majority of patients (88%) had a 1 vertebral segment involved. The median follow-up was 7 months (range, 0–47). The median age at diagnosis was 63 years (range, 41–89). Majority of the study cohort was female (69%) and Chinese ethnicity (80%). Approximately half of the patients had ECOG performance status of 0 to 1 (54%) and Charlson comorbidity index of less than 9 (60%).

**Table 1:**
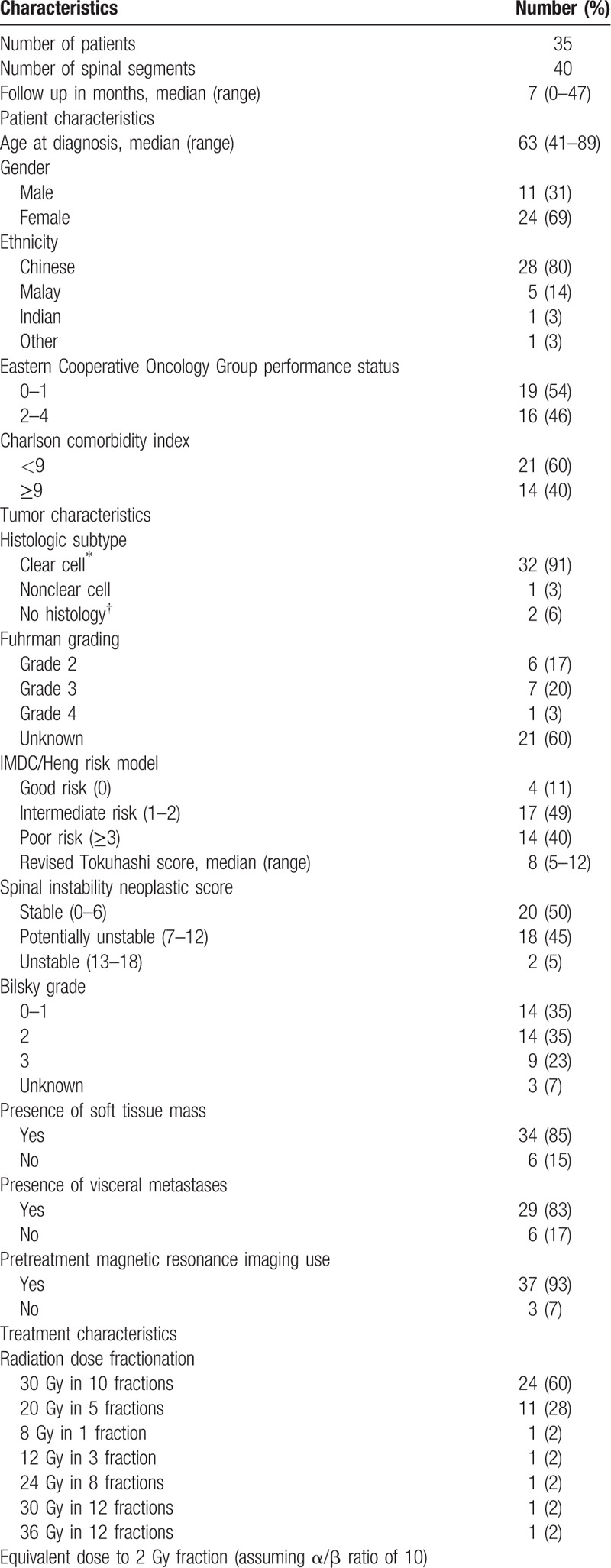
Baseline characteristics.

**Table 1 (Continued):**
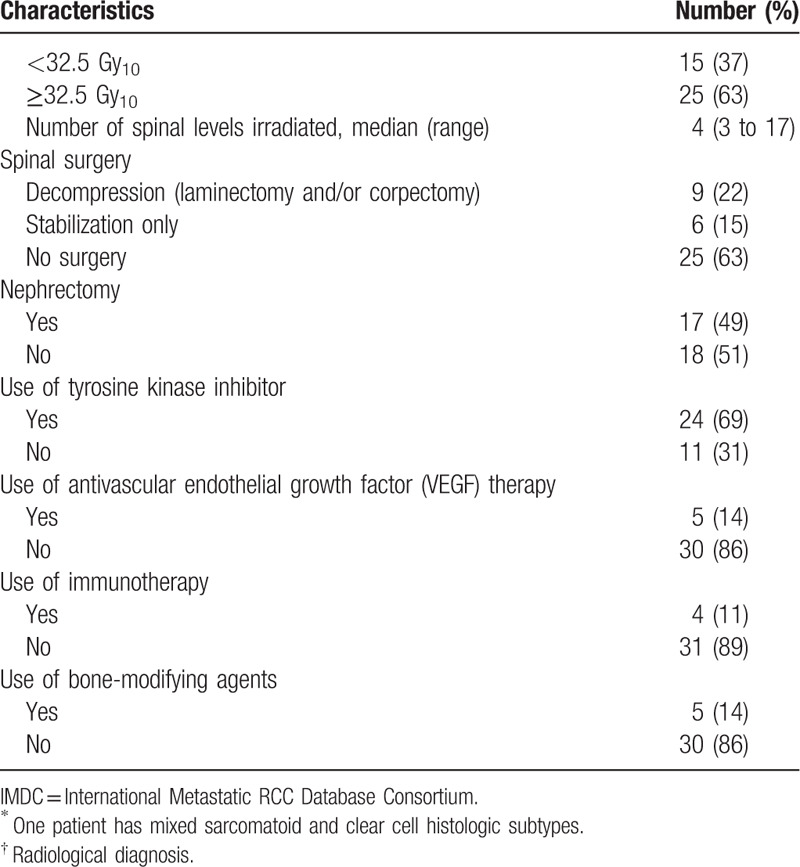
Baseline characteristics.

Histological confirmation was obtained for almost all the patients (94%). Among the patients with histology available, all but 1 patient had clear cell RCC, including 1 with mixed clear cell and sarcomatoid histologic subtype. Most of the patients had intermediate and poor risk based on IMDC risk model (89%) and estimated life expectancy of less than 6 months based on the revised Tokuhashi score (63%). Half of the patients had a SINS score of less than 7 (which indicates no spinal instability). The presence of soft tissue mass (85%) and visceral metastases (83%) were common in the study population. Pretreatment MRI was performed in nearly all the patients (93%).

The most commonly used radiation dose fractionation was 30 Gy in 10 fractions (60%) and 20 Gy in 5 fractions (28%). The median EQD_2_ was 32.5 Gy_10_ (range, 12–39). Two patients did not complete the prescribed radiation dose: 1 passed away due to gastrointestinal bleeding and another 1 was transferred to inpatient hospice. The median number of spinal levels irradiated was 4 (range, 3–17). Thirty-seven percent of them underwent surgical interventions, either decompression (22%) or stabilization only (15%). Nephrectomy was performed in half of the study population (49%). Most of the patients received tyrosine kinase inhibitors (69%); some received anti-vascular endothelial growth factor receptor (14%), immunotherapy (11%), and bone-modifying agents (14%).

### 3.2 Outcomes

Seven out of forty spinal segments irradiated (17.5%) developed local progression, with the median time to local progression of 10.2 months (range, 1.9 to 22.9). The 6- and 12-month local control rates were 97.5% and 87.5%. Three of them were re-irradiated and another one underwent surgery for local progression. At the time of last follow-up, all but 1 patient had died. The 6- and 12-month OS rates were 54.3% and 25.7%. The median local PFS and OS were 3.3 and 4.8 months.

There was no grade 3 or higher toxicity detected. Three spinal segments developed vertebral compression fracture following RT (7.5%). Pain response could not be reported due to insufficient data.

### 3.3 Univariable and multivariable analysis for local PFS and OS

**Figure 2:**
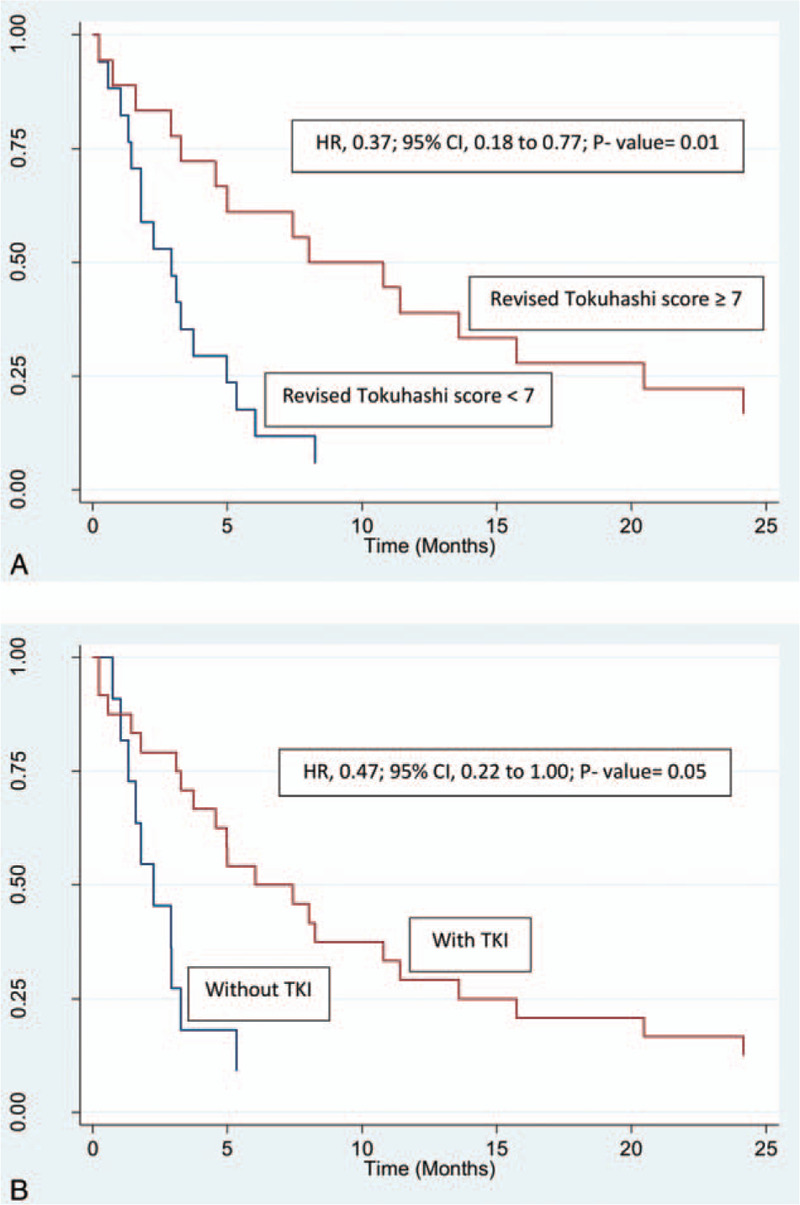
Kaplan–Meier curve for overall survival based on (A) revised Tokuhashi score (<7 vs ≥7) and (B) tyrosine kinase inhibitor use (yes vs no). CI = confidence interval, HR = hazard ratio, TKI = tyrosine kinase inhibitor.

There were no significant predictors of local PFS on multivariable analysis (Tables 2 and 4). For OS, higher revised Tokuhashi score (hazard ratio [HR], 0.41; 95% confidence interval [CI], 0.19–0.88; *P* = .02), lower number of spinal levels irradiated (HR, 1.18; 95% CI, 1.01–1.37; *P* = .04), and the use of tyrosine kinase therapy (HR, 0.41; 95% CI, 0.18–0.96; *P* = .04) were identified as the independent predictors for improved OS (Tables 3 and 4, Fig. 2A and B).

**Table 2:**
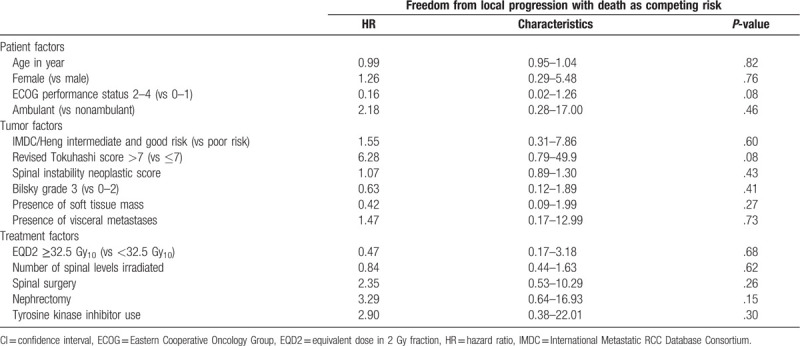
Univariable Cox proportional hazard regression: characteristics associated with local progression-free survival.

**Table 3:**
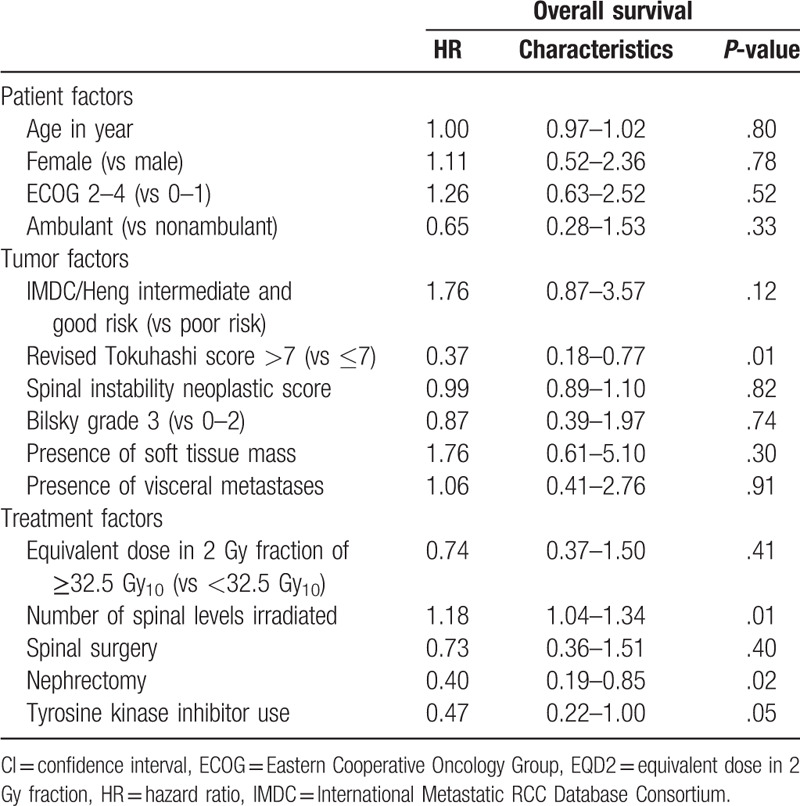
Univariable Cox proportional hazard regression: characteristics associated with overall survival.

**Table 4:**
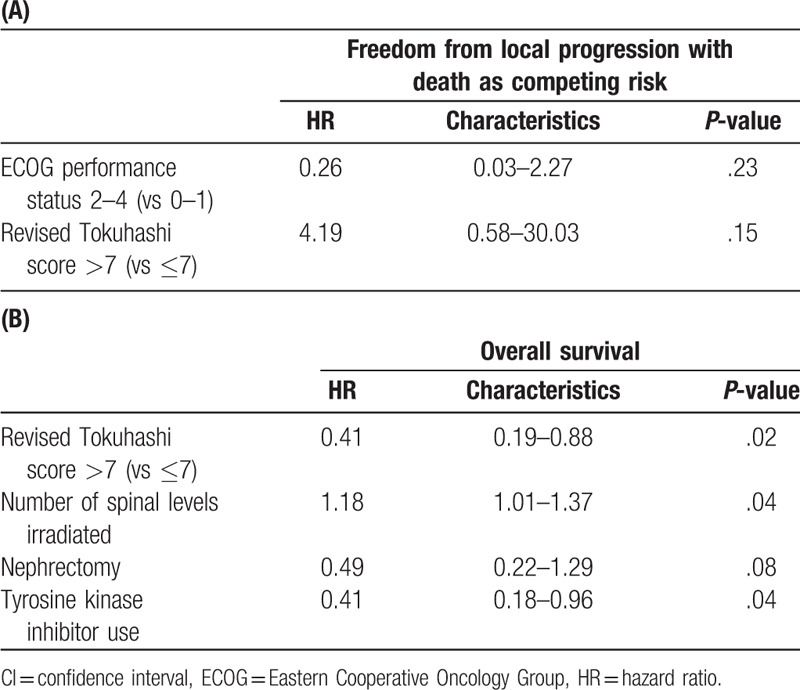
Multivariable Cox proportional hazard regression: characteristics associated with (A) local progression-free survival and (B) overall survival.

## 4 Discussion

We report the outcomes of patients with spinal metastases from RCC who were treated with cEBRT. Overall, the median survival of our cohort was relatively short at 4.8 months. The rate of local progression was low at 17.5%, which occurred at a median at 10.2 months. This suggests that most patients do not survive long enough to develop a local recurrence after palliative RT to spine metastases. Although pain response to RT was one of our endpoints, this was not uniformly captured, and; therefore, could not be reported.

The data on the outcomes of cEBRT in metastatic RCC pertaining to spine metastases is scarce.^[6,11]^ There have been a few small studies (both prospective and retrospective) which have evaluated the pain response in patients with any osseous metastases from RCC. These ranged from 60% to 80%, with the durability of pain relief ranging between 2 and 3 months.^[28–31]^ Our findings are consistent with the results reported by Ganju et al, who retrospectively analyzed the outcome of 40 patients with 53 treatment courses of palliative cEBRT (30% of patients treated with 30 Gy in 10 fractions) to any osseous metastases from RCC and reported 1-year local control rate of 62%.^[30]^ The investigators defined local control based on radiographic control as partial response, stable or progressive disease. However, it remains unclear if plain radiograph, CT imaging or MRI was used to assess response. RCC is commonly associated with soft tissue component; therefore, utilizing plain radiographs to assess response may result in false-negative findings. All these studies were largely limited by small sample size, heterogenous target population, varied treatment site and varied definition of study endpoints.

In our study, we found that higher radiation dose was not associated with improved local control. Most of our patients were treated with 30 Gy in 10 fractions, and therefore the median EQD_2_ was 32.5 Gy_10_. When compared to lower doses, such as 20 Gy in 5 fractions (EQD_2_ of 23.3 Gy_10_), we did not find a significant difference in local control rates. This was consistent with most other studies.^[30–33]^ Ganju et al analyzed biologically effective dose (BED) as a continuous variable and showed that higher BED was not significantly associated with improved pain response and radiographic control.^[30]^ Schlampp et al revealed that there was no correlation between dose fractionation and pain reduction when comparing those irradiated with more than 30 Gy and 30 Gy or less.^[31]^ In contrast, a study by DiBiase et al reported a dose-response relationship for doses above BED of 50.5 Gy_10_ (such as 39 Gy in 13 fractions).^[34]^ Overall, despite RCC having a purported radioresistant histology, inordinately high radiation doses may not be needed to achieve the goals of palliation, especially in patients where the survival is expected to be less than a year.

As mentioned above, the median OS in our study was 4.8 months (2.6 and 10.8 months for synchronous and metachronous cases, respectively). This was shorter than that reported by other studies. A systematic review reported that the median survivals of RCC patients with synchronous and metachronous spinal metastases were 7 and 11.7 months from the time of presentation.^[35]^ In our present study, higher revised Tokuhashi score and lower number of spinal levels irradiated were the independent predictors for improved OS. The number of spinal levels irradiated can be used as a proxy for the tumor burden of the patient, suggesting that patients with higher tumor burden have a worse survival. The revised Tokuhashi scoring system is widely used by spine surgeons preoperatively to estimate life expectancy, so that patients with a poor life expectancy can be spared from an aggressive intervention such as spinal surgery. This scoring system is not specific to a primary histology.^[24]^ Petteys et al validated this scoring system in 30 patients with metastatic RCC who underwent surgical intervention to the spine.^[36]^ Our findings support the use of this scoring system to estimate survival for patients with RCC undergoing palliative-intent spinal RT, with or without surgery. Fuhrman nuclear grade was identified as an independent predictor for survival in patients with RCC spinal metastases in a study of 267 patients in MD Anderson Cancer Centre and a large multicenter study of 4.063 patients by Patard et al.^[37,38]^ However, we could not demonstrate this finding in our study as majority of the patients had unknown Fuhrman grade. Besides from the revised Tokuhashi score and Fuhrman Grade, IMDC or MSKCC score is also an important scoring system for survival prognostication, though it was deemed underpowered in the previous study to detect any statistical significance in patients with RCC spinal metastases.^[35]^

Our study has several strengths. First, to the authors’ knowledge, this is the first study evaluating the outcomes of cEBRT in metastatic RCC focusing exclusively on spinal metastases. Second, this study is performed in the era where modern 3-dimensional RT techniques and targeted therapy agents are widely available, thus the study findings are more relevant and representative of the current real-world population. Thirdly, our institution adheres to a standardized radiation planning protocol and strict quality assurance. Majority of our patients were treated with either 30 Gy in 10 fractions or 20 Gy in 5 fractions. This study is limited by its small sample size and retrospective design. The data on the post-treatment follow-up was insufficiently recorded, making the assessment of pain response not feasible. In addition, the follow-up was short and limited by the short median survival of the cohort.

The implication of this study is that our findings provide important information to the spine oncology community with regards to the outcomes of cEBRT to RCC spinal metastases and justification of cEBRT being as one of the viable treatment options in this population. Despite the encouraging results on SBRT from phase II nonrandomized data,^[13–15]^ 2 retrospective studies comparing the efficacy between SBRT and cEBRT in RCC spinal metastases showed conflicting findings.^[39,40]^ We eagerly await for further direction from randomized controlled trials (RTOG 0631 and SC24 by Canadian Cancer Trials Group) which have yet to be reported.^[41,42]^ Our study highlights the need for a validated prognostication tool to guide patient selection for more intensive treatment. We also urge for future research to prospectively evaluate the outcomes of cEBRT with respect to patient-reported outcomes, imaging-based local control, and quality of life.

In conclusion, for an unselected group of patients with RCC, the incidence of local progression was low. We found no significant association between higher radiation dose (such as 30 Gy in 10 fractions) and improved local control following cEBRT to spinal metastases. This must be interpreted with caution, as this may be due to the short survival of our cohort. As the armamentarium of targeted therapy and immunotherapy continues to improve, survival will likely be prolonged. Revised Tokuhashi scoring system may be justified as a prognostication tool in the interim^[24]^; however, granular and histology-specific scoring systems are needed for patients undergoing nonoperative management. In terms of aggressive management, we continue to advocate a tailored approach for patients with a longer expected survival.

## Author contributions

**Conceptualization:** Chia Ching Lee, Timothy Cheo, Balamurugan Vellayappan.

**Data curation:** Chia Ching Lee, Timothy Cheo, Alvin Wong, Balamurugan Vellayappan.

**Formal analysis:** Jeremy Chee Seong Tey.

**Investigation:** Balamurugan Vellayappan.

**Methodology:** Chia Ching Lee, Jeremy Chee Seong Tey, Timothy Cheo, Chau Hung Lee, Alvin Wong, Balamurugan Vellayappan.

**Project administration:** Chia Ching Lee, Timothy Cheo, Naresh Kumar, Balamurugan Vellayappan.

**Resources:** Balamurugan Vellayappan.

**Software:** Chia Ching Lee, Jeremy Chee Seong Tey, Timothy Cheo, Balamurugan Vellayappan.

**Supervision:** Jeremy Chee Seong Tey, Timothy Cheo, Naresh Kumar, Balamurugan Vellayappan.

**Writing – original draft:** Chia Ching Lee, Balamurugan Vellayappan.

**Writing – review & editing:** Chia Ching Lee, Jeremy Chee Seong Tey, Timothy Cheo, Chau Hung Lee, Alvin Wong, Naresh Kumar, Balamurugan Vellayappan.

Chia Ching Lee orcid: 0000-0002-6925-8603.
